# Real-World Treatment Patterns and Cost of Care in US Ovarian Cancer Patients Undergoing BRCA Testing

**DOI:** 10.36469/001c.142444

**Published:** 2025-08-26

**Authors:** Srujitha Marupuru, Kristin Moore, Desiree Hall, Sarah Aurit, Gretchen Hultman, Noah Webb, Yong Zhu, Gieira Jones

**Affiliations:** 1 Merck; 2 Optum (United States)

**Keywords:** ovarian cancer, genetic testing, costs, treatment patterns, healthcare resource utilization

## Abstract

**Background:**

Patients with ovarian cancer incur substantial economic burdens. However, little is known about the differences in metrics such as treatment patterns, healthcare resource utilization (HCRU), and costs between those with BRCA mutant (BRCAm) and BRCA wildtype (BRCAwt) tumors.

**Objective:**

This study assessed demographic and clinical characteristics, treatment patterns, and HCRU and costs among patients diagnosed with ovarian cancer, stratified by BRCA testing status and result.

**Methods:**

This retrospective study included patients with ovarian cancer between Jan. 1, 2017, and June 30, 2022, with electronic health record (EHR) and administrative claims data in Optum’s Clinical EHR and claims databases. Data collected included baseline characteristics, lines of therapy (LOTs) (captured at 6, 12, and 24 months follow-up), HCRU (captured for 12-month baseline and follow-up periods), and costs (captured for 6-month baseline and 12-month follow-up periods). Patients were stratified by the presence or absence of a BRCA test and by BRCA testing results.

**Results:**

A total of 13 981 patients were included in the sample; 23.3% had a BRCA test and 76.7% did not. Among those with a BRCA test, 62.0% were BRCAm and 35.8% were BRCAwt. Patients who did not receive BRCA testing were more likely to be non-Hispanic African American and to live in the South (all *P* < .001). Patients who received testing were more likely to progress to a subsequent LOT but also more likely to receive BRCA-targeted therapies. The median per-patient-per-month (PPPM) total costs were 62% higher in BRCA-tested patients than those without tests (6242vs3845). Similarly, median PPPM ambulatory visits cost and pharmacy cost were 81% and 137% higher in those with BRCA tests than those without tests (2236vs1232, and 793vs335, respectively).

**Conclusions:**

Approximately one-fourth of patients received BRCA testing. Disparities existed between those who received testing and those who did not. Patients who were tested had higher costs than those who were not; this difference was driven mostly by ambulatory visits and pharmacy costs, potentially due to increased clinical encounters and higher costs of targeted treatments.

## INTRODUCTION

Ovarian cancer is the second most common cancer of the female reproductive system in the US, with 20032 new cases reported in 2021.^1^ The 5-year relative survival rate was 92% for localized invasive epithelial ovarian cancer, and 32% for distant invasive epithelial ovarian cancer, compared with the general population.^2^

Approximately 14% of ovarian cancer cases are a result of mutations in BRCA1 or BRCA2 genes.^3^ The mutations in BRCA1 and BRCA2 genes are reported to increase the risk of ovarian cancer 12-fold and 5-fold, respectively.^4^ In a meta-analysis, cumulative risk of ovarian cancer among BRCA1 and BRCA2 mutation carriers was 39% and 11% respectively, by 70 years of age.^5^ Guidelines from the American Society of Clinical Oncology (ASCO) and the National Comprehensive Cancer Network (NCCN) recommend all women diagnosed with epithelial ovarian cancer undergo germline genetic testing for BRCA1 and BRCA2 and other susceptible genes, regardless of their family history.^6,7^ Testing at the time of the initial diagnosis is recommended as results have impacts on treatment decisions.^6^ A recent study found that one-third of women with ovarian cancer underwent genetic testing; among those tested, 15% had gene variants potentially requiring a change in treatment and screening strategies.^8^ Targeted treatments such as poly-ADP ribose polymerase (PARP) inhibitors are approved by the US Food and Drug Administration (FDA) for treatment of patients with ovarian cancer with BRCA mutations.^9^ Olaparib was first approved by the FDA for the treatment of ovarian cancer in 2014; additional PARP inhibitors have since been approved.^10^

Disparities in BRCA testing rates have been documented. Lau-Min et al assessed BRCA testing rates in patients with ovarian cancer between January 2011 and March 2020.^7^ This study found that having stage 1 disease, older age, and Black or African American race were associated with a lower likelihood of BRCA testing.^7^

Yu et al examined treatment pattern among patients diagnosed with advanced ovarian cancer receiving their first line of therapy (LOT) between January 1, 2010, and December 31, 2015.^11^ Patients most frequently received platinum-/taxane-based regimens.^11^ Jorge et al examined ovarian cancer treatment patterns in patients who were BRCA mutation carriers.^12^ In this study, all patients had cytoreductive surgery and all received platinum chemotherapy.^12^ Additional real-world studies examining treatment patterns in a broader population by BRCA test status and test results are warranted.

There is a substantial economic burden in ovarian cancer. The national expenditure for ovarian cancer care was estimated to be $6.4 billion in 2020.^13^ The total cost of ovarian cancer management differed by the type of treatment. For example, a study published in 2019 reported the total cost within 8 months of ovarian cancer diagnosis varied from $105 047 for intravenous standard chemotherapy to $171 468 for regimens that included bevacizumab, while patients’ median out-of-pocket expense varied from $2511 to $4196, depending on the type of treatment and type of insurance.^14^ Simmons et al used claims data to assess healthcare resource utilization (HCRU) and costs among patients with ovarian cancer between 2010 and 2019.^13^ During treatment, ambulatory care visits were a major driver of HCRU.^13^ For patients who had at least 2 LOTs, total monthly healthcare costs were $8588 before LOT2 and increased to $15 358 during or after LOT2.^13^

There is also an increased risk of financial toxicity associated with ovarian cancer management among patients with non-White race and low income.^14^ Consequently, those vulnerable patients may have less favorable healthcare-seeking behavior, which in turn could contribute to socioeconomic inequalities in ovarian cancer mortality.^15^

While prior studies assessed treatment patterns and HCRU and costs among ovarian cancer patients, there are little data assessing disparities in BRCA tests, and whether treatment patterns, HCRU and costs differ by BRCA testing status. Similarly, little is known if these outcomes differ between those with BRCA mutant (BRCAm) and BRCA wildtype (BRCAwt) tumors. Information on these topics may provide valuable input for economic evaluation in ovarian cancer management.^16–18^ In addition, it may inform patients, healthcare professionals, and policy makers of the potential disparity in ovarian cancer treatment.

The objectives of this study were to describe demographics and clinical characteristics, treatment patterns, and HCRU and costs among patients with ovarian cancer using recent real-world data stratified by (1) those who had a BRCA test and those with no test and (2) patients with BRCAm vs BRCAwt tumors.

## METHODS

### Study Overview

This retrospective study utilized electronic health records (EHR) and administrative claims data from January 1, 2016, to December 31, 2022, to describe the healthcare journey of patients with ovarian cancer. Optum’s Clinical EHR database aggregates clinical treatment data from a network of over 140 000 providers at more than 700 hospitals and over 7000 clinics. The database is Health Insurance Portability and Accountability Act (HIPAA)–compliant and statistician-certified as de-identified. Healthcare costs were obtained from Optum’s Clinformatics® Data Mart (CDM), a research database with administrative claims data from more than 84 million patients in the US.^19^ A standard pricing algorithm was applied in CDM to account for differences in price across different plans and provider contracts. Institutional review board approval or waiver of approval was not required for this study because the study data were secondary and de-identified in accordance with the US Department of Health and Human Services Privacy Rule’s requirements for de-identification codified at 45 CFR §164.514(b). Throughout the study, patient privacy was preserved, and researchers complied strictly with all applicable HIPAA data management rules and the 1964 Helsinki Declaration and its later amendments or comparable ethical standards.

### Study Population

Included patients were female adult patients in the EHR database who had at least 2 records of *International Classification of Diseases, Ninth Revision* (ICD-9 CM) or *Tenth Revision* (ICD-10 CM) codes for ovarian cancer including cancer of fallopian tubes and peritoneum (**Supplemental Table S1**), attached to a visit, within 90 days of each other between January 1, 2017, and June 30, 2022 (identification period). The earliest record was the index date. Clinical activity of at least 1 day as evidenced by a visit record in the 12 months prior to the index date (baseline period) was required. Patients were excluded if there was evidence of other cancer during baseline. BRCA test status and results were obtained from structured variables related to biomarker, laboratory tests, or identified by Current Procedural Terminology or Healthcare Common Procedure Codes (HCPCS) (81162-81167, 81211-81217, 81432-81433, 0138U, 0129U, S3818-S3823). Patients were stratified by testing status and by BRCA result status.

### Study Variables

Demographic information collected included age as of index year, region, and race and ethnicity. Clinical characteristics captured included ovarian cancer stage from the record closest to the index date, number of metastases and metastases site within the first 3 months of the follow-up period, and body mass index (BMI).

Treatment LOTs were examined during 6, 12, and 24 months of follow-up (**Supplemental Figure S1**). Medications were captured through HCPCS and National Drug Codes (NDCs). An algorithm was used to determine LOT duration based on receipt and timing of therapy. For this algorithm, LOT capture began on the index date and all agents filled or infused within the first 30 days of LOT start comprised the regimen. LOT end was identified as the earliest of the following: initiation of a new agent, discontinuation of all agents in the regimen (gap of at least 60 days after the last day of supply), death, lack of clinical activity or end of the study period. Data captured included the 10 most common regimens in the LOTs and the proportion of patients with a subsequent LOT.

### HCRU and Costs

For HCRU analysis, data were captured from EHR data during 12 months of baseline, 12 and 24 months of follow-up. For cost analysis, data were captured from claims data and patients were required to be continuously enrolled in both pharmacy and medical coverage during a 6-month baseline and a 12-month follow-up period for inclusion in the cost analysis. HCRU and costs were calculated for ambulatory visits (physician office and hospital outpatient), emergency department (ED) visits, and inpatient (IP) admissions using methods similar to prior studies with Optum data.^13,20^ HCRU and costs were considered ovarian cancer–related if the claim had a diagnosis of ovarian cancer in the primary or secondary position or if the claim was for a treatment for ovarian cancer (either radiation, chemotherapy, or surgery). Healthcare costs were computed as per-patient per-month (PPPM) amounts. Costs were capped at 98th percentile of costs per place of service and category.

### Analysis

Descriptive statistics were used to describe the study population and results were stratified by the BRCA testing status (BRCA test vs no test) and by BRCAm vs BRCAwt status. Data were examined for normality prior to analysis. Chi-squared tests and independent sample *t*-tests were used to compare baseline characteristics. Costs were adjusted using the medical care component of the Consumer Price Index (CPI) to the year 2022. As a post-hoc analysis, multivariate analysis for associations between BRCA test status or test results and 12-month all-cause total healthcare cost in the follow-up period were examined using generalized linear models with log-link and gamma distribution, adjusting for age, race/ethnicity, region, disease stage, number of metastases, and baseline all-cause healthcare costs. Statistical analyses were conducted using SAS Enterprise Guide version 8.3.

## RESULTS

### Patient Population

A total of 13 981 patients were included; 3252 (23.3%) had a BRCA test and 10 729 (76.7%) had no BRCA test. Of those tested, 2016 (62.0%) were BRCAm and 1165 (35.8%) were BRCAwt, and 71 (2.2%) had no result (result was missing or inconclusive) (**Supplemental Figure S2**). Patients with no result were included in the test vs no test analyses but excluded from the BRCAm vs BRCAwt analyses.

The median (interquartile ranges [IQR]) follow-up time in the total study population was 18.8 (6.7-39.9) months. It was 27.9 (12.9-46.9) months and 16.0 (5.4-36.8) months for patients with and without BRCA tests, respectively; and 28.0 (13.1-47.2) months and 27.1 (12.5-46.2) months for patients with BRCAm and patients with BRCAwt, respectively (data not shown).

### Patient Characteristics

Patients with no BRCA test were more likely than those with a test to be in a young age group (18-34 years: 6.5% vs 2.2%, *P* < .001); and to be in older age groups (75-84 years: 17.1% vs 12.3%, *P* < .001; and ≥85 years 1.9% vs 0.7%, *P* < .001) (**Table 1**). Patients with a BRCA test were more likely than those with no test to be between 45-54 years old (20.2% vs 15.8%, *P* < .001) and 55-64 years old (33.4% vs 26.3%) (**Table 1**). Patients with no BRCA test were more likely to live in the South than those with a BRCA test (26.2% vs 15.3%, *P* < .001); those with a BRCA test were more likely to live in the Northeast than those with no test (25.8% vs 17.9%, *P* < .001) (**Table 1**). Patients with no test were more likely to be non-Hispanic African American than those with a test (7.8% vs 5.5%, *P* < .001) (**Table 1**).Those with no test were more likely to have stage 1 disease than those with a test (32.3% vs 18.5%, *P* < .001); those with a test were more likely than those with no test to have stage 3 disease (52.5% vs 36.5%, *P* < .001) (**Table 1**). Most characteristics examined were similar between patients with BRCAm and patients with BRCAwt.

**Table 1.** Patient Demographic and Clinical Characteristics

**Parameter**	**BRCA Test (n = 3252)**	**No BRCA Test (n = 10 729)**	***P* Value**	**Global *P* Value**	**BRCAm (n = 2016)**	**BRCAwt Negative (n = 1165)**	***P* Value**	**Global *P* Value**
Mean (SD) age, y	60.5 (12.1)	60.7 (15.0)	.392		60.0 (12.0)	61.4 (12.1)	.001	
Age group								
18-34 y	73 (2.2)	699 (6.5)	< .001	< .001	43 (2.1)	30 (2.6)	.422	<.001
35-44 y	229 (7.0)	899 (8.4)	.014		154 (7.6)	69 (5.9)	.068	
45-54 y	656 (20.2)	1693 (15.8)	< .001		444 (22.0)	199 (17.1)	< .001	
55-64 y	1087 (33.4)	2818 (26.3)	< .001		663 (32.9)	390 (33.5)	.734	
65-74 y	783 (24.1)	2581 (24.1)	.980		464 (23.0)	309 (26.5)	.026	
75-84 y	400 (12.3)	1834 (17.1)	< .001		230 (11.4)	163 (14.0)	.033	
≥85 y	24 (0.7)	205 (1.9)	< .001		18 (0.9)	5 (0.4)	.014	
Race/ethnicity, n (%)								
Non-Hispanic White	2703 (83.1)	8677 (80.9)	.004	< .001	1667 (82.7)	979 (84.0)	.328	.841
Non-Hispanic African American	178 (5.5)	836 (7.8)	< .001		115 (5.7)	58 (5.0)	.385	
Hispanics	152 (4.7)	509 (4.7)	.869		98 (4.9)	50 (4.3)	.463	
Non-Hispanic Asian	101 (3.1)	248 (2.3)	.011		63 (3.1)	37 (3.2)	.937	
Unknown	118 (3.6)	459 (4.3)	.103		73 (3.6)	41 (3.5)	.882	
Region, n (%)								
Midwest	1519 (46.7)	4613 (43.0)	< .001	< .001	930 (46.1)	563 (48.3)	.232	.117
Northeast	839 (25.8)	1920 (17.9)	< .001		549 (27.2)	268 (23.0)	.009	
South	499 (15.3)	2816 (26.2)	< .001		296 (14.7)	191 (16.4)	.196	
West	276 (8.5)	991 (9.2)	.192		167 (8.3)	100 (8.6)	.769	
Other/unknown	119 (3.7)	389 (3.6)	.929		74 (3.7)	43 (3.7)	.977	
Disease stage, n (%)^a^								
1	237 (18.5)	666 (32.3)	< .001	< .001	153 (18.4)	69 (17.0)	.548	.833
2	142 (11.1)	234 (11.3)	.837		88 (10.6)	47 (11.6)	.596	
3	671 (52.5)	752 (36.5)	< .001		436 (52.5)	219 (54.1)	.610	
4	228 (17.8)	411 (19.9)	.137		153 (18.4)	70 (17.3)	.622	
Site of metastasis, n (%)								
Visceral	1102 (33.9)	2514 (23.4)	<.001	NA^b^	706 (35.0)	369 (31.7)	.055	NA^b^
Other	751 (23.1)	1919 (17.9)	<.001		488 (24.2)	242 (20.8)	.027	
Lymph	301 (9.3)	460 (4.3)	<.001		192 (9.5)	105 (9.0)	.633	
Liver	198 (6.1)	429 (4.0)	<.001		122 (6.1)	73 (6.3)	.808	
Multiple sites^c^	82 (2.5)	191 (1.8)	.007		46 (2.3)	36 (3.1)	.166	
Lung	68 (2.1)	199 (1.9)	.389		38 (1.9)	30 (2.6)	.195	
Bone	36 (1.1)	99 (0.9)	.347		19 (0.9)	17 (1.5)	.184	
Brain	17 (0.5)	67 (0.6)	.511		8 (0.4)	9 (0.8)	.161	
No. (%) of metastases								
0	1979 (60.9)	7786 (72.6)	< .001	< .001	1204 (59.7)	732 (62.8)	.083	.331
1	579 (17.8)	1610 (15.0)	< .001		374 (18.6)	191 (16.4)	.125	
2	306 (9.4)	678 (6.3)	< .001		191 (9.5)	106 (9.1)	.726	
≥3	388 (11.9)	655 (6.1)	< .001		247 (12.3)	136 (11.7)	.629	
BMI,^d^ n (%)								
<18.5 kg/m^2^	30 (1.8)	181 (3.1)	.003	.011	19 (1.7)	11 (1.9)	.766	.449
18.5-24.9 kg/m^2^	524 (30.6)	1681 (29.0)	.205		341 (30.9)	174 (30.4)	.853	
25.0-29.9 kg/m^2^	490 (28.6)	1591 (27.5)	.347		304 (27.5)	177 (30.9)	.141	
≥30.0 kg/m^2^	668 (39.0)	2340 (40.4)	.308		441 (39.9)	210 (36.7)	.203	

Abbreviation: BMI: body mass index.

^a^Stage information was only available for 3341 of 13 981 patients.

^b^*P* value was not available because the categories were not mutually exclusive.

^c^Multiple sites include combinations of bone, brain, liver, lung, or lymph metastases.

^d^BMI information was only available for 7505 of 13 981 patients.

### Transitions Through LOTs

**Figure 1.**
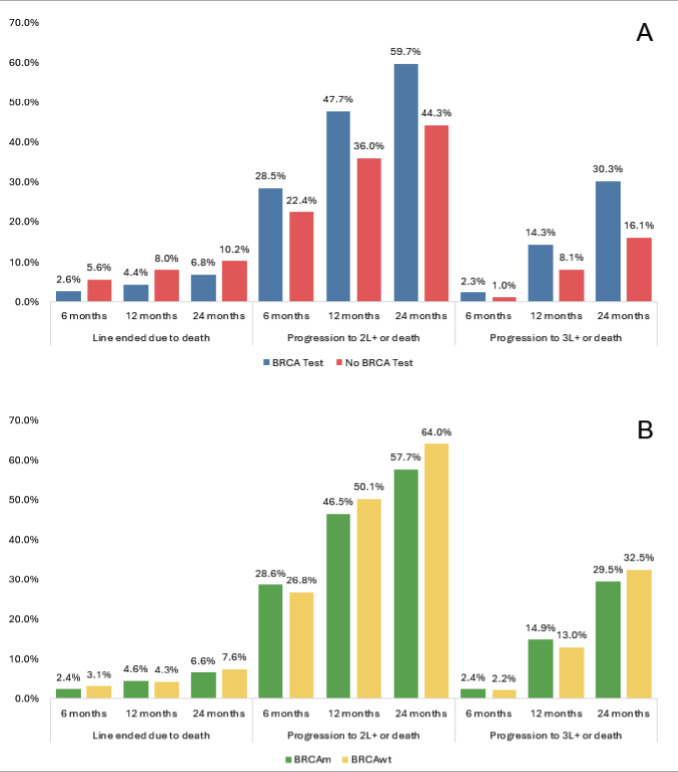
Transition Through LOTs by Number of Months in the Follow-up Period Among Patients With Ovarian Cancer Receiving Chemotherapy by (**A**) BRCA Testing Status and (**B**) BRCA Test Results Abbreviations: LOT, line of therapy; BRCA, BReast CAncer gene; BRCAm, BRCA mutant; BRCAwt, BRCA wildtype.

Among patients treated with chemotherapy, those with no BRCA test were numerically more likely to have the LOT end due to death by 24 months of follow-up than those with a test (10.2% vs 6.8%) (**Figure 1**). Patients with a BRCA test were more likely to have progressed to a later LOT (59.7% vs 44.3% progressed to LOT2; 30.3% vs 16.1% progressed to LOT3) compared with those with no test (**Figure 1**). The proportion of BRCAm and BRCAwt patients who ended a LOT due to death was similar at 24 months of follow-up (6.6% vs 7.6%) (**Figure 1**). Also, the proportion of patients who progressed to LOT2 or LOT3 appeared similar between BRCAm and BRCAwt patients.

### Treatment Regimens

**Figure 2.**
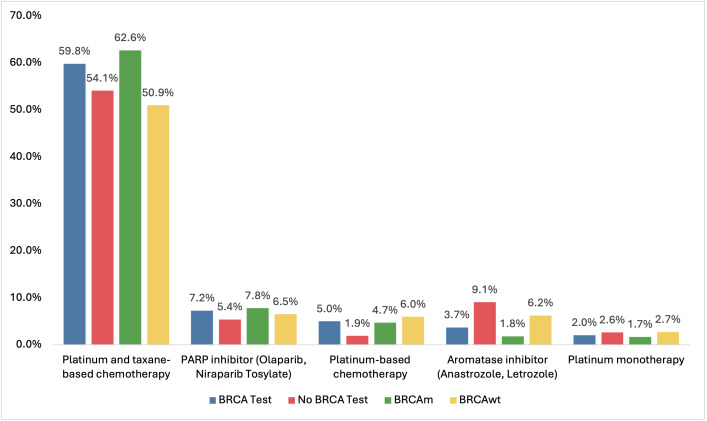
Most Common Regimens During 12 Months of Follow-up Abbreviations: BRCA, BReast CAncer gene; BRCAm, BRCA mutant; BRCAwt, BRCA wildtype; PARP, poly-ADP ribose polymerase.

Among patients treated with chemotherapy, carboplatin-paclitaxel/protein-bound was most common regimen. Patients with no test were more likely to be on aromatase inhibitors compared with those with a test (9.1% vs 3.7%) (**Figure 2**). BRCAm patients had higher PARP inhibitors use than BRCAwt patients (7.8% vs 6.5%) (**Figure 2**).

### Healthcare Resource Utilization

The median number of all-cause ambulatory visits during the baseline period was higher for those who had a BRCA test than those with no test (9.0 vs 6.0) (**Table 2**). Those who had a BRCA test had more than double the median number of ambulatory visits than those with no BRCA test within both 12 and 24 months of follow-up (25.0 vs 10.0 at 12 months and 38.5 vs 14.0 at 24 months of follow-up, respectively) (**Table 2**). This was not true for those with BRCAm vs BRCAwt (27.0 vs 24.0 at 12 months of follow-up; 39.0 vs 38.0 at 24 months of follow-up) (**Table 2**). These patterns persisted when restricted to ovarian cancer–related HCRU counts in the follow-up.

**Table 2.** All-Cause and Ovarian Cancer–related Healthcare Resource Utilization

	**Baseline (12 Months) **					**12 Months Follow-up**					**24 Months Follow-up**				
**Total **	**BRCA Test **	**No BRCA Test **	**BRCAm**	**BRCAwt **	**Total **	**BRCA Test **	**No BRCA Test **	**BRCAm**	**BRCAwt**	**Total **	**BRCA Test **	**No BRCA Test **	**BRCAm**	**BRCAwt**	
All-cause healthcare utilization counts, median (IQR)															
ED visits	1.0 (1-2)	1.0 (1-2)	1.0 (1-2)	1.0 (1-2)	1.0 (1-2)	1.0 (1-2)	2.0 (1-3)	1.0 (1-2)	2.0 (1-3)	2.0 (1-3)	2.0 (1-3)	2.0 (1-3)	2.0 (1-3)	2.0 (1-3)	2.0 (1-3)
IP stay	1.0 (1-1)	1.0 (1-1)	1.0 (1-1)	1.0 (1-1)	1.0 (1-1)	1.0 (1-2)	1.0 (1-2)	1.0 (1-2)	1.0 (1-2)	1.0 (1-2)	1.0 (1-2)	2.0 (1-3)	1.0 (1-2)	2.0 (1-3)	2.0 (1-3)
Ambulatory visits	6.0 (2-⁠15)	9.0 (3-⁠23)	6.0 (2-⁠13)	9.0 (3-⁠21)	11.0 (3-⁠26)	12.0 (5-⁠29)	25.0 (11-⁠49)	10.0 (4-⁠22)	27.0 (11-⁠49)	24.0 (10-⁠49)	17.0 (6-⁠41)	38.5 (17-⁠74)	14.0 (5-⁠31)	39.0 (17-⁠74)	38.0 (15-⁠74)
Ovarian cancer–related healthcare utilization counts, median (IQR)															
ED visits	1.0 (1-1)	1.0 (1-1)	1.0 (1-1)	1.0 (1-1)	1.0 (1-1)	1.0 (1-2)	1.0 (1-2)	1.0 (1-2)	1.0 (1-2)	1.0 (1-2)	1.0 (1-2)	1.0 (1-2)	1.0 (1-2)	1.0 (1-2)	1.0 (1-2)
IP stay	1.0 (1-1)	1.0 (1-1)	1.0 (1-1)	1.0 (1-1)	1.0 (1-1)	1.0 (1-2)	1.0 (1-2)	1.0 (1-2)	1.0 (1-2)	1.0 (1-2)	1.0 (1-2)	1.0 (1-3)	1.0 (1-2)	1.0 (1-3)	1.0 (1-3)
Ambulatory visits	1.0 (1-3)	2.0 (1-9)	1.0 (1-2)	1.0 (1-7)	3.0 (1-13)	4.0 (2-14)	12.0 (4-26)	3.0 (1-9)	13.0 (4-27)	10.0 (3-25)	5.0 (2-18)	16.0 (5-38)	4.0 (2-11)	18.0 (5-39)	15.0 (5-38)

Abbreviations: BRCA, BReast CAncer gene; BRCAm, BRCA mutant; BRCAwt, BRCA wildtype; ED, emergency department; IP, inpatient; IQR, interquartile range.

### Costs

The median total PPPM all-cause healthcare costs during the baseline period were higher among those with a test compared with those with no test ($1248 vs $1094) (**Table 3**). Patients who had a BRCA test had higher median all-cause PPPM total costs within 12 months of diagnosis compared with those with no BRCA test ($6242 vs $3845) (**Table 3**). The median PPPM pharmacy costs were higher for those with a BRCA test than for those with no test ($793 vs $335). Those with BRCAm had higher median all-cause costs associated with IP stays ($1593 vs $1) and pharmacy costs ($1222 vs $409) than BRCAwt patients (**Table 3**). All-cause PPPM total costs were higher in BRCAm patients compared with BRCAwt patients ($9297 vs $5255) (**Table 3**). Similarly, ovarian cancer–related PPPM total costs in the 12-month follow-up period were higher in patients with a BRCA test compared with those with no test and for BRCAm patients compared with BRCAwt patients.

**Table 3.** All-Cause and Ovarian Cancer–related Healthcare Costs, Per Patient Per Month

	**Baseline (6 Months) **					**12 Months Follow-up**				
**Total**	**BRCA Test**	**No BRCA Test**	**BRCAm**	**BRCAwt**	**Total**	**BRCA Test**	**No BRCA Test**	**BRCAm**	**BRCAwt**	
All-cause healthcare costs in US dollars,^a^ PPPM Median (IQR)										
Ambulatory	639 (234-1509)	765 (416-1588)	584 (183-1485)	871 (534-1802)	638 (165-1229)	1506 (503-3283)	2236 (944-4177)	1232 (407-3072)	2091 (1086-4215)	2399 (592-3504)
ED	0 (0-70)	0 (0-8)	0 (0-96)	0 (0-25)	0 (0-0)	0 (0-125)	0 (0-105)	0 (0-129)	0 (0-116)	0 (0-49)
Inpatient	0 (0-0)	0 (0-0)	0 (0-30)	0 (0-0)	0 (0-0)	37 (0-3467)	174 (0-3747)	17 (0-3057)	1593 (0-3843)	1 (0-3468)
Pharmacy	71 (12-397)	111 (13-455)	66 (12-362)	118 (16-334)	74 (8-536)	402 (56-2447)	793 (126-3894)	335 (47-1852)	1222 (261-5521)	409 (39-2690)
Other	0 (0-0)	0 (0-11)	0 (0-0)	0 (0-0)	0 (0-16)	0 (0-33)	0 (0-139)	0 (0-32)	0 (0-138)	0 (0-70)
Total	1106 (442-⁠4823)	1248 (510-⁠3976)	1094 (395-⁠5405)	1457 (708-⁠3621)	899 (437-⁠4577)	4379 (1286-⁠9980)	6242 (2532-⁠13114)	3845 (1031-⁠8827)	9297 (2714-⁠13259)	5255 (951-⁠11828)
Ovarian cancer–related healthcare costs in US dollars,^a^ PPPM Median (IQR)										
Ambulatory	0 (0-247)	21 (0-547)	0 (0-139)	19 (0-369)	18 (0-886)	591 (33-2203)	1425 (349-3170)	247 (19-1970)	1556 (468-3282)	1160 (193-2813)
ED	0 (0-0)	0 (0-0)	0 (0-0)	0 (0-0)	0 (0-0)	0 (0-0)	0 (0-0)	0 (0-0)	0 (0-0)	0 (0-0)
Inpatient	0 (0-0)	0 (0-0)	0 (0-0)	0 (0-0)	0 (0-0)	0 (0-1745)	0 (0-3344)	0 (0-1128)	195 (0-3448)	0 (0-1662)
Pharmacy	0 (0-0)	0 (0-0)	0 (0-0)	0 (0-0)	0 (0-0)	0 (0-242)	65 (0-2360)	0 (0-156)	85 (0-3539)	18 (0-694)
Other	0 (0-0)	0 (0-0)	0 (0-0)	0 (0-0)	0 (0-0)	0 (0-0)	0 (0-0)	0 (0-0)	0 (0-0)	0 (0-11)
Total	0 (0-577)	25 (0-702)	0 (0-494)	41 (0-444)	18 (0-1513)	1675 (85-5175)	4031 (434-8696)	956 (38-4160)	4384 (936-9885)	2618 (320-6564)

Abbreviations: BRCA, BReast CAncer gene; BRCAm, BRCA mutant; BRCAwt, BRCA wildtype; CPI, Consumer Price Index; ED, emergency department; PPPM, per patient per month.

^a^Adjusted for CPI 2022 US dollars.

### Associations Between BRCA Test Status and Total Healthcare Costs

**Table 4** presents associations between BRCA test status and 12-month all-cause total healthcare costs in the follow-up period in ovarian cancer patients, adjusting for age, race/ethnicity, region, disease stage, metastasis, and baseline all-cause total healthcare costs. There was no significant association between BRCA test status and 12-month all-cause total healthcare costs (cost ratio for patients with BRCA test vs no test = 1.116, 95% confidence interval [CI] = [0.882, 1.413], *P* = .360).

**Table 4.** Generalized Linear Model of All-Cause Healthcare Costs in the 12-Month Follow-up Period by BRCA Test Status or BRCA Test Results

	**Cost Ratio**	**Lower 95% CI**	**Upper 95% CI**	***P* Value**	**Predicted Value ($)**
**BRCA test status**					
Intercept				<.001	
Cohort					
BRCA test	1.116	0.882	1.413	.360	7428
No BRCA test	Ref.				6655
Age	0.992	0.985	0.999	.026	
Race/ethnicity					
Non-Hispanic White	Ref.				
Non-Hispanic African American	0.587	0.399	0.862	.007	
Hispanics	1.009	0.652	1.563	.967	
Non-Hispanic Asian	1.737	0.864	3.491	.121	
Unknown	1.517	0.673	3.417	.315	
Region					
Midwest	Ref.				
Northeast	0.721	0.538	0.964	.028	
South	0.961	0.746	1.237	.755	
West	0.917	0.660	1.274	.605	
Other/unknown	1.050	0.617	1.788	.857	
Disease stage					
1	Ref.				
2	1.371	0.725	2.591	.332	
3	1.478	0.897	2.435	.126	
4	1.952	0.987	3.862	.055	
Unknown	0.835	0.567	1.228	.360	
No. of metastases					
0	Ref.				
1	1.462	1.080	1.978	.014	
2	1.578	1.064	2.342	.023	
≥3	1.659	1.069	2.577	.024	
Baseline all-cause healthcare costs (quartiles)					
Q1 (0−440)	Ref.				
Q2 (441−1094)	1.473	1.114	1.947	.007	
Q3 (1095−4823)	1.769	1.335	2.342	<.001	
Q4 (4824−36 607)	2.359	1.773	3.139	<.001	
**BRCA test results**					
Intercept				<.001	
Cohort					
BRCAm	1.010	0.700	1.458	.957	8711
BRCAwt	Ref.				8623
Age	0.998	0.983	1.014	.836	
Race/ethnicity					
Non-Hispanic White	Ref.				
Non-Hispanic African American	0.448	0.189	1.061	.068	
Hispanics	1.425	0.694	2.926	.334	
Non-Hispanic Asian	0.654	0.217	1.974	.451	
Unknown	0.490	0.071	3.357	.467	
Region					
Midwest	Ref.				
Northeast	1.276	0.744	2.186	.376	
South	1.102	0.681	1.782	.692	
West	1.090	0.581	2.046	.788	
Other/unknown	1.327	0.503	3.503	.568	
Disease stage					
1	Ref.				
2	0.561	0.199	1.581	.274	
3	2.240	1.000	5.020	.050	
4	1.937	0.704	5.330	.201	
Unknown	1.136	0.539	2.395	.737	
No. of metastases					
0	Ref.				
1	1.138	0.660	1.962	.643	
2	1.618	0.956	2.737	.073	
≥3	2.029	1.068	3.857	.031	
Baseline all-cause healthcare costs (quartiles)					
Q1 (0−503)	Ref.				
Q2 (504−1161)	2.396	1.404	4.088	.001	
Q3 (1162−3852)	2.340	1.323	4.137	.003	
Q4 (3853−23 033)	3.186	1.890	5.372	<.001	

Abbreviations: BRCA, BReast CAncer gene; CI, confidence interval.

### Associations Between BRCA Test Results and Total Healthcare Costs

**Table 4** presents associations between BRCA test results and 12-month all-cause total healthcare costs in the follow-up period in ovarian cancer patients, adjusting for age, race/ethnicity, region, disease stage, metastasis, and baseline all-cause total healthcare costs. There was no significant association between BRCA test results and 12-month all-cause total healthcare costs (cost ratio for patients with BRCAm vs BRACwt = 1.010, 95% CI = [0.700, 1458], *P* = .957).

## DISCUSSION

Study findings indicate that approximately one-fourth of patients received BRCA testing and there are underlying differences in demographic and clinical characteristics of patients who received testing compared with those who did not. Those who received BRCA testing were more likely to move through LOTs than those who did not receive testing. Those with a test had nearly 2.5 times the median number of PPPM ambulatory visits than those with no BRCA. Patients who had a test had higher median all-cause PPPM total costs; the median PPPM pharmacy costs were more than doubled for those who had a BRCA test than for those with no test.

The rate of BRCA testing reported in this study is lower than the rates reported in other published studies.^7,8^ Because this study relied only on structured EHR data for BRCA tests, there is a potential for underreporting of testing; a test may have not been documented in the EHR or a patient may have had a panel test and the result of the BRCA gene may not have been clearly documented. Additionally, the study period included the COVID-19 pandemic, which may have resulted in lower rates of testing and testing delays.^21^ Lastly, all stages of ovarian cancer were included in this study population. However, the FDA approval of PARP inhibitor (olaparib) for frontline maintenance therapy in ovarian cancer was in December 2018, and testing in frontline may not be broadly adopted until later. This may contribute to the lower testing rate during the study period in the present study.

The prevalence of BRCAm in an unselected ovarian cancer population has been estimated to be 12% to 14%.^22^ In this study, 62% of patients among those who had BRCA tests were found to be mutation positive. This percentage should not be interpreted as prevalence of BRCA mutation rate in ovarian cancer patients because of several reasons. First, the study population was highly selective. For example, patients were required to have 12 months of lookback activity before the index date with at least 1 record of diagnosis within 90 days of the earliest diagnosis. This means patients needed to have frequent clinical visits recorded in the EHR database, which could reflect a severe disease condition with mutation that required frequent clinical encounters. Second, the denominator, which is the number of patients with BRCA tests, could have been underestimated; patients who had negative mutation results could be more likely to have no mention of the test and test results in their clinical notes compared with those who had positive results. These could result in a lower percentage of patients with negative mutation among patients who had BRCA test records in the EHR data.

Patients who did not receive a BRCA test were more likely to be non-Hispanic African American than those who did. This aligns with the work of Lau-Min et al, who reported that Black or African American race was associated with a lower likelihood of BRCA testing in ovarian cancer patients.^7^ We also observed differences in region, with those who did not have a test being more likely to live in the South than those who did, which could be due in part to other demographic differences. Additionally, there were differences in age, with patients with a BRCA test being more likely than those with no test to be between 45 and 64 years old. There were also differences in stage; those with no test were more likely to have stage 1 disease than those with a test and those with a test were more likely than those with no test to have stage 3 disease. This also aligns with the work of Lau-Min et al, who reported that those with stage 1 disease were less likely than those with advanced disease to receive testing.^7^

One difference observed in treatment patterns is that those who received BRCA testing were more likely to move through LOTs than those who did not. Notably, this study did not ascertain the timing of BRCA testing in relation to LOTs; it is possible that after receiving testing patients were transitioning to a different LOT based on test results or were receiving testing after progression or failing a regimen. Patients who received BRCA testing are potentially getting different types of treatment than those who did not. BRCA test results are recommended to be used to determine which maintenance therapy is appropriate for patients following standard first-line chemotherapy.^23^

Those who had a BRCA test had more than double the number of PPPM ambulatory visits than those with no BRCA test within 12 and 24 months after diagnosis. These patients also had higher median all-cause PPPM total costs after diagnosis than those who had no BRCA test. These findings could signal higher engagement by patients and care teams among those who had a BRCA test. This increase in visits could be used to help patients stay up-to-date on disease management,^24^ discuss options and implications for secondary genomic findings,^25^ and point to more appointments with specialists such as genetic counselors. In fact, in addition to recommending genetic testing, ASCO, the US Preventative Services Task Force, the NCCN, the Society of Gynecologic Oncology, and the American College of Obstetrics and Gynecologists also recommend universal genetic counseling.^26^ Median PPPM pharmacy costs were more than doubled for those who had a BRCA test than for those with no test. This could potentially be due to the higher cost of targeted treatments, which were more prevalent in this population. PARP inhibitors can be costly. Liang *et al* estimated the cost of PARP inhibitors among commercially insured ovarian patients in the MarketScan database between 2014 and 2017 and found that the median total cost was $13 342.^27^

The post-hoc analysis revealed no significant association between BRCA test status or test results and total healthcare cost, when adjusted for demographic and clinical characteristics of patients. While similar studies on associations between BRCA test and healthcare costs were not found in the literature, a previous study^28^ examined predictors of high costs in ovarian cancer patients and identified factors such as lower income and use of open procedures were significantly associated with higher healthcare costs in ovarian cancer patients. The lack of significant association in the present study is likely because of insufficient statistical power due to the small sample sizes available for the cost analysis. Future studies with a large sample size should be considered.

The low rate of BRCA tests among patients with ovarian cancer in the present study highlighted the gap between clinical guidelines and clinical practice. Healthcare professionals were the most common source for genetic test information for ovarian cancer patients^29^; therefore, healthcare professionals are encouraged to discuss with patients about genetic tests. This could be a useful approach for increased awareness and possibly higher utilization of genetic tests. In addition, policy makers may consider strategies for increased accessibility and reduced financial burden of genetic tests in ovarian cancer care, for example, through increased coverage of genetic tests in health plans.

There are several limitations which must be considered when interpreting these findings. The Optum EHR database is an open data ecosystem, and some clinical information including BRCA tests may be unobserved if patients seek care outside the system. The number of patients with BRCA mutation status available was relatively small, potentially limiting the generalizability of the findings. Information on the type of BRCA testing that a patient received (germline vs somatic) was not available; testing guidelines are different for different types of testing makes it difficult to know if guidelines were followed. This study reports the total percentage of patients receiving BRCA testing; it is possible that some patients who did not receive testing were not eligible. Analyses were not able to ascertain the timing of BRCA testing in the patient’s care journey, which limits interpretation. The HCRU and cost analysis required continuous coverage for 12 months in the follow-up period; this may have introduced a selection bias, as patients who had poor prognosis, potentially with higher cost per month, could die within 12 months. Moreover, the association between BRCA test status or test results and healthcare cost may not indicate any causal relationship, and there could be potential residue confounding as certain information, such as socioeconomic status of patients, was not available. Future studies could consider administration of patient surveys to collect self-reported information such as education, income, and information for measurement of indirect cost; moreover, the survey data may be linked with real-world data to provide a complete assessment of economic burden. Finally, data related to homologous recombination deficiency testing was not assessed.

## CONCLUSIONS

Not all ovarian cancer patients received BRCA testing, and there were disparities in testing status. Patients who received BRCA testing were more likely to progress to a higher LOT but were also more likely to receive therapies that are targeted to the underlying genomics of their cancer. Higher costs were seen in patients who were BRCA tested and BRCAm and this difference is driven mostly by pharmacy costs.

### Ethical Conduct of Research Statement

Throughout the study, patient privacy was preserved, and researchers complied strictly with all applicable Health Insurance Portability and Accountability Act data management rules and the 1964 Helsinki Declaration and its later amendments or comparable ethical standards.

## Supplementary Material

Online Supplementary Material

## Data Availability

The data contained in our database contains proprietary elements owned by Optum and therefore cannot be broadly disclosed or made publicly available at this time. The disclosure of these data to third-party clients assumes certain data security and privacy protocols are in place and that the third-party client has executed our standard license agreement, which includes restrictive covenants governing the use of the data.
