# Supplementary material for: Real-World Treatment Patterns and Cost of Care in US Ovarian Cancer Patients Undergoing BRCA Testing
Source: J Health Econ Outcomes Res. 2025 Aug 26;12(2):85–97. doi: 10.36469/001c.142444 (PMC12393876; doi:10.36469/001c.142444)
Supplement: Online Supplementary Material [file jheor_2025_12_2_142444_299678.pdf]

JHEOR

Journal of Health Economics and Outcomes Research

Online Supplementary Material

Real-World Treatment Patterns and Cost of Care in US Ovarian Cancer Patients Undergoing BRCA Testing. *JHEOR*. 2025;12(2):85-97. doi:10.36469/jheor.2025.142444

Figure S1: Study Diagram

Figure S2: Patient Sample Selection

Table S1: ICD-9 CM and ICD-10 CM Codes for Ovarian Cancer

This supplementary material has been provided by the authors to give readers additional information about their work.

This is an open-access article distributed under the terms of the Creative Commons Attribution 4.0 International License (CCBY-4.0). View this license's legal deed at http://creativecommons.org/licenses/by/4.0 and legal code at http://creativecommons.org/licenses/by/4.0/legalcode for more information.

Figure S1. Study Diagram

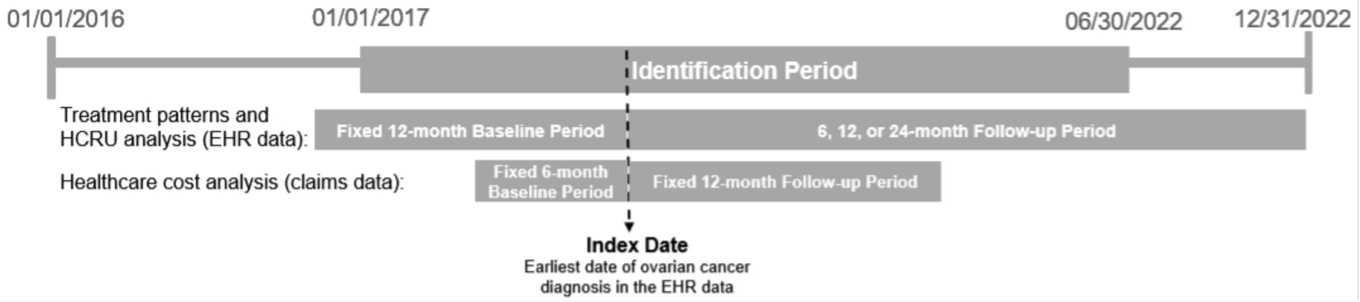

Abbreviations: BRCA, BReast Cancer gene; EHR, electronic health records.

Figure S2. Patient Sample Selection

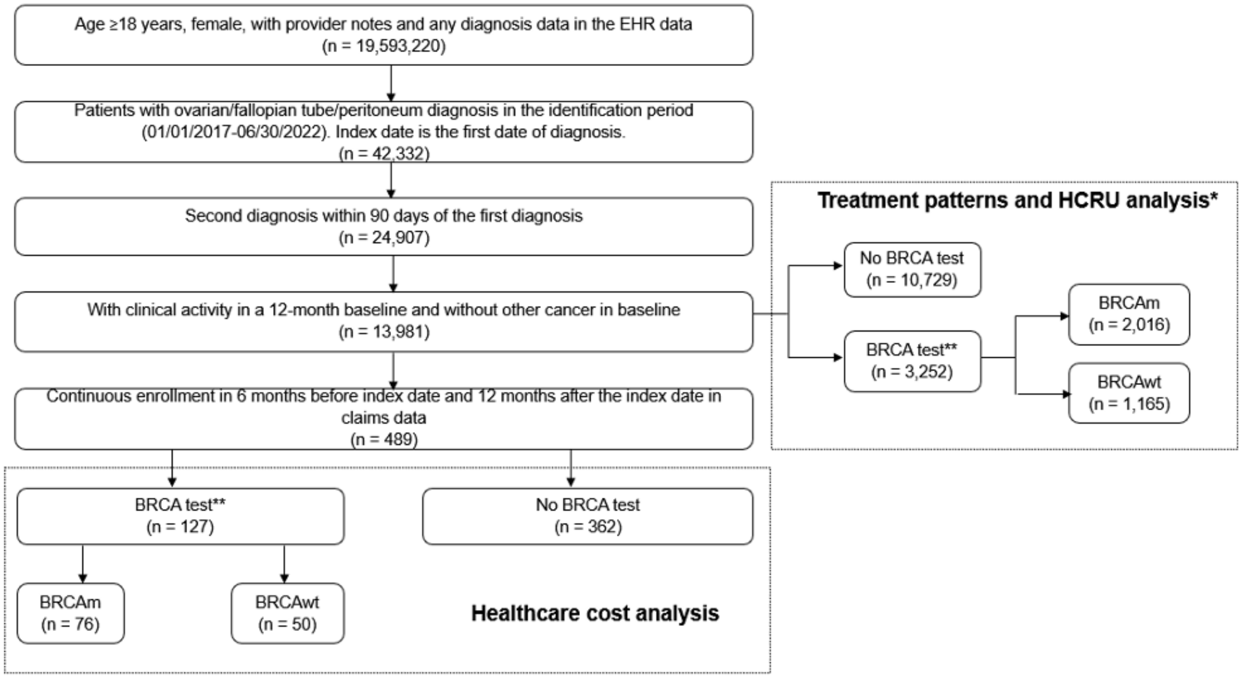

*Sample size presented for characteristics of patients. Final sample size varied depending on the length of follow-up examined.
**Patients with a BRCA test but with missing or invalid results were not included in the analysis by BRCA test results.

Abbreviations: BRCA, BReast Cancer gene; EHR, electronic health records.

Table S1. ICD-9 CM and ICD-10 CM Codes for Ovarian Cancer

ICD-9 CM
158.8
158.9
183.0
183.2
ICD-10 CM
C48.1
C48.2
C56
C56.1
C56.9
C56.9
C57.0
C57.00
C57.01
C57.02

Abbreviations: ICD-9,-CM, *International Classification of Diseases, Ninth Revision*; ICD-10-CM, I*nternational Classification of Diseases, Tenth Revision*.
